# Impact of coronavirus pandemic and containment measures on HIV diagnosis

**DOI:** 10.1017/S0950268820001867

**Published:** 2020-08-24

**Authors:** Gilles Darcis, Dolores Vaira, Michel Moutschen

**Affiliations:** 1 Infectious Diseases Department, Liège University Hospital, Liège, Belgium; 2 AIDS Reference Laboratory, Liège University, Liège, Belgium

**Keywords:** COVID-19 pandemic, HIV diagnosis, HIV screening

## Abstract

During the last months and following the implementation of containment measures in the context of coronavirus disease 2019 (COVID-19) pandemic, the number of new human immunodeficiency virus (HIV) diagnoses radically decreased in Liege AIDS Reference Center, Belgium. The number of HIV screening tests has also dramatically dropped down to an unprecedented level. This decline of HIV diagnosis is caused by missed diagnoses of individuals infected before the establishment of such measures and to the reduction of high-risk sexual behaviours during the COVID-19 pandemic.

## Impact of COVID-19 pandemic and containment measures on HIV diagnosis

The coronavirus disease 2019 (COVID-19) pandemic caused by severe acute respiratory syndrome coronavirus 2 (SARS-CoV-2) has already affected more than 20 million people in more than 200 countries and territories and resulted in more than 700 000 deaths (https://gisanddata.maps.arcgis.com/apps/opsdashboard/index.html#/bda7594740fd40299423467b48e9ecf6). Most countries around the world have implemented a range of community containment strategies to prevent transmission of SARS-CoV-2. Health care systems have been dramatically changed by the crisis with the worthy objectives of facing ingoing flows of infected patients, reducing nosocomial transmission and protecting health care providers. COVID-19 has also become the top differential diagnosis in any person with flu-like symptoms, leading to a rapid isolation of at-risk patients with testing of SARS-CoV-2. Altogether, those practices eventually mitigate the outbreak in several countries in Europe, including Belgium (https://www.sciensano.be/en).

The first SARS-CoV-2 transmissions in Belgium were described at the beginning of March 2020. The pandemic then rapidly increased, making Belgium one of the world's worst affected countries in terms of the number of deaths per capita. Belgian authorities gradually implemented containment measures from 13 March (e.g. closure of schools, discos, restaurants and the cancellation of all public gatherings). Additional measures were imposed from 18 March with penalties for individuals who did not respect the restrictions. Citizens were required to stay at home to avoid contact outside of their family, except for essential travel (e.g. to the doctor, food shops, pharmacy).

The coronavirus pandemic and containment measures dramatically affected the Belgian health care system, including human immunodeficiency virus (HIV) screening. In our centre of Liege University Hospital, Belgium, a mean of 8.4 new diagnoses has been made monthly during the last 15 months (Table 1). About 1000 HIV screening tests are performed every month (Table 1). However, since the beginning of the SARS-CoV-2 pandemic and the implementation of strict containment measures, the number of HIV screening tests and diagnoses dramatically decreased (Table 1).

**Table 1.** Number of HIV screening tests and HIV new diagnoses, per month, in Liege University Hospital

Month	Number of HIV screening tests	Number of HIV diagnoses
January-19	1161	13
February-19	1026	8
March-19	1011	7
April-19	943	11
May-19	996	7
June-19	979	5
July-19	972	7
August-19	874	13
September-19	978	13
October-19	1108	8
November-19	1025	9
December-19	970	9
January-20	1046	6
February-20	925	8
March-20	634	10
**Median (IQR)**	**979 (943 ; 1026)**	**8 (7 ; 11)**
April-20	306	2
May-20	497	2
**Median (IQR)**	401* (306 ; 497)	2** (2 ; 2)

Poisson regression.

* *P* < 0.0001.

** *P* = 0.0032.

This is likely a multifactorial phenomenon. The rapid decline of HIV diagnoses, which directly followed the beginning of the SARS-CoV-2 pandemic, is most likely caused by missed diagnoses of individuals infected before the establishment of such measures. In addition to well-known barriers to HIV screening (e.g. fear, stigma), the SARS-CoV-2 pandemic was indeed the cause of additional hurdles to HIV testing at different levels.

From an individual perspective, fear of getting COVID-19 at the hospital or in facilities where HIV screening test can be done is one of them. Testing for HIV would also add more stress to an already stressful situation. Moreover, the symptoms related to HIV infection could have been misinterpreted as caused by SARS-CoV-2. High rates of missed opportunities for HIV diagnosis have previously been reported, highlighting the ongoing need for physician education on HIV testing and clinical signs suggestive of HIV infection [1, 2]. This is likely to be exacerbated in the context of COVID-19 pandemic.

From an organisational perspective, the reduction of public transport and the work overload in infectious diseases units as well as virology laboratories could have created other barriers to HIV testing.

At the community and societal level, SARS-CoV-2 infection has been in the spotlight, while communication about other diseases including HIV has been poorly perceptible.

Finally, a reduction of high-risk sexual behaviours associated with containment measures such as closing of gathering places has possibly contributed to the decrease of new HIV diagnoses.

In conclusion, the SARS-CoV-2 pandemic and associated containment measures will result in a transient decrease of HIV incidence. However, it could also favour late diagnosis, an issue that was already described before the dramatic events we are currently dealing with [1, 2].

Given the importance of rapid HIV diagnosis, public health communications should highlight that effective HIV care is readily and safely available despite SARS-CoV-2 pandemic, that early diagnosis of HIV improves health outcomes, and that HIV infection can mimic other infectious diseases including SARS-CoV-2 infection.

## Availability of data and materials

All data generated or analysed during this study are included in this published article.
